# It is time to define an organizational model for the prevention and management of infections along the surgical pathway: a worldwide cross-sectional survey

**DOI:** 10.1186/s13017-022-00420-4

**Published:** 2022-03-17

**Authors:** Massimo Sartelli, Francesco M. Labricciosa, Federico Coccolini, Raul Coimbra, Fikri M. Abu-Zidan, Luca Ansaloni, Majdi N. Al-Hasan, Shamshul Ansari, Philip S. Barie, Miguel Angel Caínzos, Marco Ceresoli, Massimo Chiarugi, Jeffrey A. Claridge, Enrico Cicuttin, Evan Patchen Dellinger, Donald E. Fry, Xavier Guirao, Timothy Craig Hardcastle, Andreas Hecker, Ari K. Leppäniemi, Andrey Litvin, Sanjay Marwah, Emilio Maseda, John E. Mazuski, Ziad Ahmed Memish, Andrew W. Kirkpatrick, Leonardo Pagani, Mauro Podda, Huseyin Kemal Rasa, Boris E. Sakakushev, Robert G. Sawyer, Fabio Tumietto, Yonghong Xiao, Wedad Faraj Aboubreeg, Harissou Adamou, Lali Akhmeteli, Emrah Akin, Maria Grazia Alberio, Felipe Alconchel, Ibrahim Amadou Magagi, Ana Belén Araúz, Giulio Argenio, Boyko C. Atanasov, Semra Demirli Atici, Selmy Sabry Awad, Efstratia Baili, Lovenish Bains, Miklosh Bala, Oussama Baraket, Suman Baral, Vladislav A. Belskii, Moussa Benboubker, Offir Ben-Ishay, Pierpaolo Bordoni, Abdalia Boumédiène, Giuseppe Brisinda, Laura Cavazzuti, Sujith J. Chandy, Maria Michela Chiarello, Nicola Cillara, Guglielmo Clarizia, Maria-Elena Cocuz, Iuliu Gabriel Cocuz, Luigi Conti, Raffaella Coppola, Yunfeng Cui, Jacek Czepiel, Fabrizio D’Acapito, Dimitrios Damaskos, Koray Das, Belinda De Simone, Samir Delibegovic, Zaza Demetrashvili, Dzemail S. Detanac, Sameer Dhingra, Stefano Di Bella, Evgeni N. Dimitrov, Agron Dogjani, Mario D’Oria, Irina Magdalena Dumitru, Mutasim M. Elmangory, Octavian Enciu, Massimo Fantoni, Daniela Filipescu, Francesco Fleres, Domitilla Foghetti, Pietro Fransvea, Mahir Gachabayov, Rita Galeiras, Gianni Gattuso, Wagih M. Ghannam, Valeria Ghisetti, Giorgio Giraudo, Kebebe Bekele Gonfa, Emre Gonullu, Yousif Tag Elsir Y. Hamad, Matthias Hecker, Arda Isik, Nizar Ismail, Azzain Ismail, Sumita Agarwal Jain, Souha S. Kanj, Garima Kapoor, Ilias Karaiskos, Alfie J. Kavalakat, Jakub Kenig, Faryal Khamis, Vladimir Khokha, Ronald Kiguba, Jae Il Kim, Yoshiro Kobe, Kenneth Yuh Yen Kok, Bojan M. Kovacevic, Igor Andreevich Kryvoruchko, Akira Kuriyama, Aitor Landaluce-Olavarria, Konstantinos Lasithiotakis, Varut Lohsiriwat, Eftychios Lostoridis, Davide Luppi, Gustavo Miguel Machain Vega, Marc Maegele, Athanasios Marinis, Gennaro Martines, Aleix Martínez-Pérez, Damien Massalou, Cristian Mesina, Gökhan Metan, María Guadalupe Miranda-Novales, Shyam Kumar Mishra, Mohaned Ibrahim Hussein Mohamed, Ali Yasen Y. Mohamedahmed, Ismael Mora-Guzmán, Francesk Mulita, Ana-Maria Musina, Pradeep H. Navsaria, Ionut Negoi, Gabriela Elisa Nita, Donal B. O’Connor, Carlos Alberto Ordoñez, Desiré Pantalone, Arpád Panyko, Aristeidis Papadopoulos, Nikolaos Pararas, Francesco Pata, Tapan Patel, Gianluca Pellino, Teresa Perra, Gennaro Perrone, Antonio Pesce, Tadeja Pintar, Georgi Ivanov Popivanov, Alberto Porcu, Martha Alexa Quiodettis, Razrim Rahim, Ashrarur Rahman Mitul, Martin Reichert, Miran Rems, Glendee Yolande Reynolds Campbell, Nuno Rocha-Pereira, Gabriel Rodrigues, Gustavo Eduardo Roncancio Villamil, Stefano Rossi, Ibrahima Sall, Hossein Samadi Kafil, Diego Sasia, Jeremiah Seni, Charalampos Seretis, Mario Serradilla-Martín, Vishal G. Shelat, Boonying Siribumrungwong, Mihail Slavchev, Leonardo Solaini, Boun Kim Tan, Antonio Tarasconi, Dario Tartaglia, Elena Adelina Toma, Gia Tomadze, Adriana Toro, Marcos Roberto Tovani-Palone, Harry van Goor, Alin Vasilescu, Andras Vereczkei, Massimiliano Veroux, Sergio Alberto Weckmann, Lukas Werner Widmer, AliIbrahim Yahya, Sanoop K. Zachariah, Andee Dzulkarnaen Zakaria, Nadezhda Zubareva, Wietse P. Zuidema, Isidoro Di Carlo, Francesco Cortese, Gian Luca Baiocchi, Ronald V. Maier, Fausto Catena

**Affiliations:** 1 Department of Surgery, Macerata Hospital, Macerata, Italy; 2 Global Alliance for Infections in Surgery, Macerata, Italy; 3 General, Emergency and Trauma Surgery Department, Pisa University Hospital, Pisa, Italy; 4 Comparative Effectiveness and Clinical Outcomes Research Center, Riverside University Health System Medical Center, Riverside, USA; 5 Department of Surgery, College of Medicine and Health Sciences, UAE University, Al-Ain, United Arab Emirates; 6 Department of Surgery, Fondazione IRCCS Policlinico San Matteo, University of Pavia, Pavia, Italy; 7 Department of Internal Medicine, University of South Carolina School of Medicine, Columbia, USA; 8 Department of Microbiology, Chitwan Medical College and Teaching Hospital, Bharatpur, Chitwan Nepal; 9 Department of Surgery, Weill Cornell Medicine, New York, USA; 10 Department of Surgery, University Hospital, Santiago de Compostela, Spain; 11 Department of General and Emergency Surgery, Milano-Bicocca University, School of Medicine and Surgery, Milan, Italy; 12 MetroHealth Medical Center, Case Western Reserve University, Cleveland, OH USA; 13 Department of Surgery, University of Washington, Seattle, USA; 14 Department of Surgery, Northwestern University Feinberg School of Medicine, Chicago, USA; 15 Surgical Endocrine Head and Neck Unit, Department of General Surgery, Parc Tauli, Hospital Universitari, Sabadell, Spain; 16 Trauma and Burn Service, Department of Surgery, University of KwaZulu-Natal, Durban, South Africa; 17 Department of Surgery, University Hospital of Giessen, Giessen, Germany; 18 Abdominal Surgery, Helsinki University Hospital and University of Helsinki, Helsinki, Finland; 19 Department of Surgical Disciplines, Immanuel Kant Baltic Federal University, Regional Clinic Hospital, Kaliningrad, Russia; 20 Department of Surgery, BDS Post-Graduate Institute of Medical Sciences, Rohtak, India; 21 Surgical Critical Care, Department of Anesthesia, Hospital Valdecilla, Santander, Spain; 22 Department of Surgery, Washington University in Saint Louis, Saint Louis, USA; 23 Research and Innovation Center, King Saud Medical City, College of Medicine, Alfaisal University, Riyadh, Kingdom of Saudi Arabia; 24 General, Acute Care, Abdominal Wall Reconstruction, and Trauma Surgery, Foothills Medical Centre, Calgary, Canada; 25 Antimicrobial Stewardship Program, Bolzano Central Hospital, Bolzano, Italy; 26 Department of Emergency Surgery, Cagliari University Hospital “D. Casula”, AOU Cagliari, Cagliari, Italy; 27 Department of Surgery, Anadolu Medical Center, Kocaeli, Turkey; 28 General Surgery, UMHAT St George Plovdiv, RIMU/Research Institute at Medical University of Plovdiv, Plovdiv, Bulgaria; 29 Department of Surgery, Homer Stryker, M.D., School of Medicine, Western Michigan University, Kalamazoo, USA; 30 Infectious Diseases Unit, IRCCS Azienda Ospedaliero Universitaria di Bologna, Bologna, Italy; 31 State Key Laboratory for Diagnosis and Treatment of Infectious Diseases, The First Affiliated Hospital, School of Medicine, Zhejiang University, Hangzhou, China; 32 Department of Surgery, Zliten Medical Center, Zliten, Libya; 33 Department of Surgery, University of Zinder, Zinder, Niger; 34 Department of Surgery, TSMU First University Clinic, Tbilisi, Georgia; 35 Department of Surgery, Sakarya University Educational and Research Hospital, Sakarya, Turkey; 36 Department of Surgery, Circolo Hospital and Macchi Foundation, Varese, Italy; 37 Department of Surgery and Transplantation, Virgen de La Arrixaca University Hospital, El Palmar, Spain; 38 Infectious Diseases Unit, Hospital Santo Tomás, Panama City, Panama; 39 Emergency Surgery Unit, AOU San Giovanni di Dio e Ruggi d’Aragona, University of Salerno, Salerno, Italy; 40 Department of Surgery, UMHAT Eurohospital Plovdiv, RIMU/Research Institute at Medical University of Plovdiv, Plovdiv, Bulgaria; 41 Department of Surgery, University of Health Sciences Tepecik Training and Research Hospital, Izmir, Turkey; 42 Department of Trauma and Acute Care Surgery, Mansoura University Hospital, Mansoura University, Mansoura, Egypt; 43 Second Department of Surgery, IASO General Hospital, Athens, Greece; 44 Department of Surgery, Maulana Azad Medical College, New Delhi, India; 45 Department of Surgery, Hadassah Hebrew University Medical Center, Jerusalem, Israel; 46 Department of Surgery, Department of Surgery, Bizerte Hospital, Tunis El Manar University, Tunis, Tunisia; 47 Department of Surgery, Dirghayu Pokhara Hospital, Pokhara, Nepal; 48 Department of Anesthesiology and Intensive Care, Privolzhskiy District Medical Center, Nizhny Novgorod, Russia; 49 HAIs Control Committee, HASSAN II University Hospital Fez, Fez, Morocco; 50 Department of Surgery, Rambam Health Care Campus, Haifa, Israel; 51 Department of Surgery, ASST Valtellina e Alto Lario, Sondrio Hospital, Sondrio, Italy; 52 Clinique Mutualiste de La Porte de L’Orient, Lorient, France; 53 Medical and Surgical Sciences Department, Fondazione Policlinico Universitario “A. Gemelli” IRCCS, Rome, Italy; 54 Medical Directorate, Arcispedale Santa Maria Nuova Hospital, Local Health Authority-IRCSS of Reggio Emilia, Reggio Emilia, Italy; 55 Department of Pharmacology and Clinical Pharmacology, Christian Medical College, Vellore, India; 56 Department of Surgery, Ospedale San Giovanni di Dio, Azienda Sanitaria Provinciale, Crotone, Italy; 57 Department of Surgery, PO Santissima Trinità ASSL Cagliari, Cagliari, Italy; 58 Faculty of Medicine, University Transilvania of Brasov, Brasov, Romania; 59 Pathophysiology Department, George Emil Palade University of Medicine, Pharmacy, Sciences and Technology, George Emil Palade of Targu Mures, Targu Mures, Romania; 60 Department of Surgery, Ospedale G. Da Saliceto, AUSL Piacenza, Piacenza, Italy; 61 Unit of Microbiology, Betania Hospital, Naples, Italy; 62 Department of Surgery, Tianjin Nankai Hospital, Nankai Clinical School of Medicine, Tianjin Medical University, Tianjin, China; 63 Department of Infectious and Tropical Diseases, Jagiellonian University Medical College, Krakaw, Poland; 64 Department of Surgery, Forlì Morgagni-Pierantoni Hospital, AUSL Romagna, Forlì, Italy; 65 Department of Surgery, Royal Infirmary of Edinburgh, Edinburgh, UK; 66 Department of Surgery, University of Health Sciences, Adana City Training and Research Hospital, Adana, Turkey; 67 Emergency and Metabolic Minimally Invasive Surgery, Poissy-Saint-Germain-en-Laye Hospital, Yvelines, France; 68 Department of Surgery, University Clinical Center Tuzla, Tuzla, Bosnia and Herzegovina; 69 Department of Surgery, Kipshidze Central University Hospital, Tbilisi, Georgia; 70 Department of Surgery, General Hospital Novi Pazar, Novi Pazar, Serbia; 71 Department of Pharmacy Practice, National Institute of Pharmaceutical Education and Research (NIPER), Hajipur, Bihar India; 72 Clinical Department of Medical, Surgical and Health Sciences, Trieste University, Trieste, Italy; 73 Department of Surgical Diseases, University Hospital “Prof. Dr. Stoyan Kirkovich”, Stara Zagora, Bulgaria; 74 Department of Surgery, University of Medicine of Tirana, Tirana, Albania; 75 Division of Vascular and Endovascular Surgery, Cardiovascular Department, University Hospital of Trieste, Trieste, Italy; 76 Clinical Infectious Diseases Hospital, Ovidius University, Constanta, Romania; 77 Epidemiology Unit, National Public Health Laboratory, Khartoum, Sudan; 78 Department of Surgery, Carol Davila University of Medicine and Pharmacy, Bucharest, Romania; 79 Dipartimento Di Scienze Di Laboratorio E Infettivologiche, Fondazione Policlinico Gemelli IRCCS, Rome, Italy; 80 Cardiac Anaesthesia and Intensive Care 2, Emergency Institute of Cardiovascular Diseases, University of Medicine and Pharmacy Carol Davila, Bucharest, Romania; 81 Department of Surgery, Azienda Ospedaliera Marche Nord, Pesaro, Italy; 82 Department of Abdominal Surgery, Vladimir City Emergency Hospital, Vladimir, Russia; 83 Critical Care Unit, Complexo Hospitalario Universitario, La Coruna, Spain; 84 Department of Infectious Diseases, Carlo Poma” Hospital ASST, Mantova, Italy; 85 Department of Surgery, Mansoura Faculty of Medicine, Mansoura, Egypt; 86 Laboratory of Microbiology and Virology, Amedeo di Savoia Hospital and ASL Città di Torino, Turin, Italy; 87 Department of Surgery, Aso Santa Croce e Carle, Cuneo, Italy; 88 Department of Surgery, Madda Walabu University Goba Referral Hospital, Bala-Robe, Ethiopia; 89 Department of Surgery, Sakarya University, Adapazarı, Turkey; 90 Department of Surgery, East Surrey Hospital, Redhill, UK; 91 Medical Clinic II, University Hospital Giessen, Glessen, Germany; 92 Department of Surgery, Istanbul Medeniyet University, Istanbul, Turkey; 93 Trauma and Orthopaedics, Stoke Mandeville Hospital, Aylesbury, UK; 94 Trauma and Orthopaedics Woodend Hospital, Aberdeen, UK; 95 Department of Surgery, SMS Medical College and Hospital, Adarsh, India; 96 Infectious Diseases Division, American University of Beirut Medical Center, Beirut, Lebanon; 97 Department of Microbiology, Gandhi Medical College, Bhopal, India; 98 1St Department of Internal Medicine-Infectious Diseases, Hygeia Hospital, Marousi, Greece; 99 Department of Surgery, Jubilee Mission Medical College and RI, Thrissur, India; 100 Department of General Surgery, Jagiellonian University Medical College, Kraków, Poland; 101 Infectious Diseases and Internal Medicine Department, Royal Hospital, Muscat, Oman; 102 Department of Emergency Surgery, City Hospital, Mozyr, Belarus; 103 Pharmacology and Therapeutics, College of Health Sciences, Makerere University, Kampala, Uganda; 104 Department of Surgery, Inje University Ilsan Paik Hospital, Goyang, South Korea; 105 Department of Surgery, Chiba Emergency Medical Center, Chiba, Japan; 106 Discipline of Medicine, Pengiran Anak Puteri Rashidah Sa’adatul Bolkiah Institute of Health Sciences, Brunei Darussalam University, Darussalam, Brunei; 107 Department of Surgery, UMC Zvezdara, Belgrade, Serbia; 108 Surgery No. 2 Unit, Kharkiv National Medical University, Kharkiv, Ukraine; 109 Emergency and Critical Care Center, Kurashiki Central Hospital, Kurashiki, Japan; 110 Department of Surgery, Urduliz-Alfredo Espinosa Hospital, Urduliz, Spain; 111 Department of Surgery, University Hospital of Heraklion, Heraklion, Greece; 112 Department of Surgery, Faculty of Medicine Siriraj Hospital, Mahidol University, Bangkok, Thailand; 113 1St Department of Surgery, Kavala General Hospital, Kavala, Greece; 114 Department of Surgery, ASMN IRCCS, Reggio Emilia, Italy; 115 General Surgery, Universidad Nacional de Asunción-Facultad de Ciencias Medicas, Hospital de Clínicas, Asuncion, Paraguay; 116 Department of Trauma and Orthopedic Surgery, Cologne-Merheim Medical Center (CMMC), University Witten/Herdecke, Cologne, Germany; 117 Third Department of Surgery, Tzaneio General Hospital, Piraeus, Greece; 118 Department of Surgery, Azienda Ospedaliero Universitaria Policlinico, Bari, Italy; 119 Department of General and Digestive Surgery, Hospital Universitario Doctor Peset, Valencia, Spain; 120 Acute Care Surgery, Centre Hospitalier Universitaire de Nice, Nice University Hospital, Nice, France; 121 Department of Surgery, Emergency County Hospital of Craiova, Craiova, Romania; 122 Infectious Diseases and Clinical Microbiology, Hacettepe University Faculty of Medicine, Ankara, Turkey; 123 Infectious Diseases Department, Paediatric Hospital, Analysis and Synthesis Research Unit, Social Security Mexican Institute, Mexico City, Mexico; 124 Clinical Microbiology, Tribhuvan University Teaching Hospital, Institute of Medicine, Kathmandu, Nepal; 125 Plastic Surgery, Leeds General Infirmary, Leeds, UK; 126 Department of Surgery, Sandwell and West, Birmingham Hospitals NHS Trust, Lyndon, UK; 127 Department of Surgery, Hospital General La Mancha Centro, Alcazar de San Juan, Spain; 128 Department of Surgery, General University Hospital of Patras, Rio, Greece; 129 Department of Surgery, University of Medicine and Pharmacy Grigore T Popa, Iasi, Romania; 130 Trauma Centre, Groote Schuur Hospital and University of Cape Town, Cape Town, South Africa; 131 Department of Surgery, Emergency Hospital of Bucharest, Carol Davila University of Medicine and Pharmacy, Bucharest, Romania; 132 Department of Surgery, Trinity College, Dublin, Ireland; 133 Division of Trauma and Acute Care Surgery, Fundacion Valle del Lili, Cali, Colombia; 134 Emergency Surgery Department, AOU Careggi-Università di Firenze, Florence, Italy; 135 IVth Department of Surgery, Faculty of Medicine, Comenius University, Bratislava, Slovakia; 136 Surgical Department, General Hospital of Nikaia, Nikaia, Greece; 137 Surgical Department, Dr. Sulaiman Al Habib Hospital, Alfaisal University, Riyadh, Saudi Arabia; 138 Department of Surgery, Nicola Giannettasio Hospital, Corigliano-Rossano, Italy; 139 Department of Surgery, Baroda Medical College, Vadodara, India; 140 Department of Advanced Medical and Surgical Sciences, Università degli Studi della Campania “Luigi Vanvitelli”, Naples, Italy; 141 Department of Medical, Surgical and Experimental Sciences, University of Sassari, Azienda Ospedaliero Universitaria di Sassari, Sassari, Italy; 142 Department of Emergency Surgery, Parma University Hospital, Parma, Italy; 143 Department of Surgery, Azienda USL of Ferrara-University of Ferrara, Ferrara, Italy; 144 Abdominal Surgery Department, UMC Ljubljana, Ljubljana, Slovenia; 145 Department of Surgery, Military Medical Academy, Sofia, Bulgaria; 146 Department of Surgery, Hospital Santo Tomás, Panama City, Panama; 147 Department of Surgery, Universiti Sains Islam Malaysia, Nilai, Malaysia; 148 Unit of Pediatric Surgery, Bangladesh Shishu Hospital and Institute, Dhaka, Bangladesh; 149 Department for General and Abdominal Surgery, General Hospital Jesenice, Jesenice, Slovenia; 150 Department of Microbiology, University Hospital of the West Indies, Kingston, Jamaica; 151 Centro Hospitalar E Universitário São João, Porto, Portugal; 152 Department of Surgery, Kasturba Medical College, Manipal Academy of Higher Education, Manipal, India; 153 Department of Medicine, Division of Infectious Diseases, Universidad Pontificia Bolivariana, Medellín, Colombia; 154 Research Group on Cardiovascular and Pulmonary Diseases, Clínica Cardio VID, Medellín, Colombia; 155 Emergency Surgery Department, San Filippo Neri Hospital, Rome, Italy; 156 Department of Surgery, Military Teaching Hospital, Hôpital Principal de Dakar, Dakar, Senegal; 157 Drug Applied Research Center, Faculty of Medicine, Tabriz University of Medical Sciences, Tabriz, Iran; 158 Department of Microbiology and Immunology, Weill Bugando School of Medicine, Catholic University of Health and Allied Sciences, Mwanza, Tanzania; 159 Department of Surgery, George Eliot Hospital NHS Trust, Nuneaton, UK; 160 Department of Surgery, Instituto de Investigación Sanitaria Aragón, Miguel Servet University Hospital, Zaragoza, Spain; 161 Department of Surgery, Tan Tock Seng Hospital, Novena, Singapore; 162 Department of Surgery, Thammasat University Hospital, Khlong Luang, Thailand; 163 Department of Surgery, University Hospital Eurohospital, Plovdiv, Bulgaria; 164 Department of Medical and Surgical Sciences, University of Bologna, Forlì, Italy; 165 Infection Prevention and Control Unit, Centre des Massues, French Red Cross, Lyon, France; 166 Department of Surgery, Tbilisi State Medical University, Tbilisi, Georgia; 167 Department of General Surgery, E. Muscatello Augusta Hospital, Augusta, Italy; 168 Department of Pathology and Legal Medicine, Ribeirão Preto Medical School, University of São Paulo, Ribeirão Preto, Brazil; 169 Department of Surgery, Radboud University Medical Center, Nijmegen, The Netherlands; 170 Department of Surgery, St. Spiridon University Hospital “Grigore T Popa” University of Medicine and Pharmacy, Iasi, Romania; 171 Department of Surgery, Medical Center University of Pécs, Pécs, Hungary; 172 Department of Medical and Surgical Sciences and Advanced Technologies, University of Catania, Catania, Italy; 173 Coordinación de Cirugía, Instituto Mexicano del Seguro Social, Mexico City, Mexico; 174 Department of Visceral Surgery and Medicine, Bern University Hospital, Bern, Switzerland; 175 Department of Surgery, Zliten Medical Center, Zilten, Libya; 176 Department of Surgery, Kerala Institute of Medical Sciences, Thiruvananthapuram, Kerala India; 177 Department of Surgery, School of Medical Sciences and University Hospital Sains Malaysia, Sains Malaysia University, Penang, Malaysia; 178 Department of General Surgery, Perm State Medical University N.a. Academician E.A. Wagner, Perm, Russia; 179 Department of Surgery, Amsterdam University Medical Center, Amsterdam, The Netherlands; 180 Emergency Surgery Department, San Filippo Neri Hospital, ASL Roma 1, Rome, Italy; 181 Department of Surgery, AAST Cremona, Cremona, Italy; 182 Department of Clinical and Experimental Sciences, University of Brescia, Brescia, Italy; 183 Harborview Medical Center, Department of Surgery, University of Washington, Seattle, WA USA; 184 Department of Surgery, Bufalini” Hospital, Cesena, Italy

**Keywords:** Cross-sectional survey, Antimicrobial stewardship, Antibiotic prescribing, Antibiotic resistance, Infection prevention and control

## Abstract

**Background:**

The objectives of the study were to investigate the organizational characteristics of acute care facilities worldwide in preventing and managing infections in surgery; assess participants’ perception regarding infection prevention and control (IPC) measures, antibiotic prescribing practices, and source control; describe awareness about the global burden of antimicrobial resistance (AMR) and IPC measures; and determine the role of the Coronavirus Disease 2019 pandemic on said awareness.

**Methods:**

A cross-sectional web-based survey was conducted contacting 1432 health care workers (HCWs) belonging to a mailing list provided by the Global Alliance for Infections in Surgery. The self-administered questionnaire was developed by a multidisciplinary team. The survey was open from May 22, 2021, and June 22, 2021. Three reminders were sent, after 7, 14, and 21 days.

**Results:**

Three hundred four respondents from 72 countries returned a questionnaire, with an overall response rate of 21.2%. Respectively, 90.4% and 68.8% of participants stated their hospital had a multidisciplinary IPC team or a multidisciplinary antimicrobial stewardship team. Local protocols for antimicrobial therapy of surgical infections and protocols for surgical antibiotic prophylaxis were present in 76.6% and 90.8% of hospitals, respectively. In 23.4% and 24.0% of hospitals no surveillance systems for surgical site infections and no monitoring systems of used antimicrobials were implemented. Patient and family involvement in IPC management was considered to be slightly or not important in their hospital by the majority of respondents (65.1%). Awareness of the global burden of AMR among HCWs was considered very important or important by 54.6% of participants. The COVID-19 pandemic was considered by 80.3% of respondents as a very important or important factor in raising HCWs awareness of the IPC programs in their hospital. Based on the survey results, the authors developed 15 statements for several questions regarding the prevention and management of infections in surgery. The statements may be the starting point for designing future evidence-based recommendations.

**Conclusion:**

Adequacy of prevention and management of infections in acute care facilities depends on HCWs behaviours and on the organizational characteristics of acute health care facilities to support best practices and promote behavioural change. Patient involvement in the implementation of IPC is still little considered. A debate on how operationalising a fundamental change to IPC, from being solely the HCWs responsibility to one that involves a collaborative relationship between HCWs and patients, should be opened.

**Supplementary Information:**

The online version contains supplementary material available at 10.1186/s13017-022-00420-4.

## Background

Improving patient safety in health care requires a systematic approach to combat antimicrobial resistance (AMR) and prevent and treat infections appropriately. The concepts go hand-in-hand [1]. AMR has emerged as one of the major problems of public health worldwide, resulting in a crisis of global proportions that threatens the modern practices of medicine and animal health, as well as food security [2]. Although a natural phenomenon that occurs as bacteria evolve, human activities have accelerated the pace at which bacteria develop and propagate AMR. The threat of AMR represents one of the most significant patient safety challenges of our time.

Multi-drug-resistant organisms (MDROs) in humans, animals, or the environment may spread between species and from one country to another [2]. Despite the complexity of the problem, health care workers (HCWs) play a crucial role in preventing the emergence and spread of AMR.

Hospitalized patients may have multiple risk factors for AMR acquisition, and acute care facilities are incubators for their development [1]. The intensity of patient care in acute care facilities creates an environment that facilitates both the emergence and transmission of AMR [3]. In these settings, excessive and inappropriate use of antibiotics and poor infection prevention and control (IPC) practices are the two main drivers of AMR. Moreover, despite evidence supporting best practices in preventing and managing infections, evidence-based practices are often underused in routine practice. Surgical infections constitute a global burden of disease. Development of surveillance, infection prevention and control (IPC), and antimicrobial stewardship (AS)s are initial steps forward. Education is critical and should begin early in training, be an active process and be sustained through regular programs [4].

Prevention and management of infections along the surgical pathway should always focus on collaboration among HCWs and sharing knowledge of best practices [5, 6]. Some of the most common clinical conditions that surgeons manage are infectious. Additionally, health care-associated infections (HAIs), such as surgical site infections (SSIs), catheter-associated urinary tract infections (CA-UTIs), and hospital-acquired or ventilator-associated bacterial pneumonia (HABP/VABP), are among the most common complications surgeons face in clinical practice.

IPC is a pivotal component of all health systems, affecting the health and safety of patients. An established culture of safe health care practices can prevent and control the dissemination of pathogens and is crucial for containing the spread of AMR, because every infection prevented equates to one less instance of antibiotic use. Thus, IPC is one of the main pillars of the global framework to reduce AMR [7].

HAIs are the most common adverse events in health care. Although HAIs are common, the global burden remains unknown because of the difficulty in gathering reliable data worldwide. Patients at particular risk of HAIs are those who undergo surgical procedures and patients with medical devices (e.g. central lines, urinary catheters, and ventilators). HAIs result in substantial morbidity and mortality, necessitate additional diagnostic and therapeutic interventions, prolong hospital stay, and generate additional cost. However, the importance of the phenomenon is not yet sufficiently perceived among HCWs, resulting in a poor level of responsiveness when it comes to prevention [6]. Considering that HAIs are often-preventable, HAIs are considered a patient safety issue and an indicator of the quality of patient care [8]. Moreover, many HAIs are caused by MDROs such as methicillin-resistant *Staphylococcus aureus* (MRSA), vancomycin-resistant *Enterococcus* (VRE), extended-spectrum beta-lactamase (ESBL)-producing Gram-negative bacilli, or carbapenemase-producing Gram-negative bacilli (CRE). IPC plays a pivotal role by preventing HAIs, assisting with prompt detection of MDROs and promoting compliance with standard and transmission-based precautions. In fact, effective prevention tactics reduce the incidence of MDROs, minimize HAIs, and decrease antibiotic use.

Antibiotics can be life-saving when treating bacterial infections but are often used inappropriately (e.g. drug choice, duration, or dosing). Many HCWs underestimate the burden of AMR in their hospitals, and thus, many antibiotics prescribed are unnecessary or prescribed incorrectly.

By far, the most important reason for inappropriate prescribing practices in hospitals is lack of knowledge, but cultural and social reasons may also play a role. Systematic approaches to optimizing antibiotic use worldwide are now urgently necessary [1].

Antimicrobial stewardship (AS) is a pivotal component of IPC. AS promotes the appropriate use of antibiotics, improving patients’ outcomes and decreasing the incidence of infections caused by MDROs. Several studies demonstrated that ASPs significantly reduce the incidence of infections and colonization with MDROs and *Clostridium difficile* infections of hospital inpatients [9]. The best tactical approaches for effective AS programs are not definitively established and are likely to vary based on local culture, policies, routine clinical practice, and probably on resources. Many hospitals remain without formal programs, and those that maintain as programs continue to struggle with gaining acceptance across service lines. Therefore, every hospital worldwide should utilize existing resources to create an effective multidisciplinary AS program. AS’ policies should be based on international/national antibiotic prescribing guidelines and tailored to local microbiology and AMR patterns. Based on guidelines and local formulary options promoted by the AS team, facility-specific treatment recommendations can guide clinicians in antibiotic agent selection and duration of therapy. Because physicians are the primary prescribers of antimicrobial agents, education to change attitudes and improve knowledge directed towards physicians are crucial for improving antibiotic prescribing practices.

Finally, source control (SC) encompasses all physical measures undertaken to eliminate the source of infection, ideally during the index procedure. As a general principle, every verified source of infection should be controlled as soon as possible. Appropriate SC is of utmost importance in the management of surgical infections. Intra-abdominal infections and soft tissue infections are the types where SC is most feasible and most likely to be impactful, but even then, can be ineffective initially in as many as one-quarter of cases. SC, when effective, reduces the bacterial inoculum and corrects or controls any anatomic derangements to restore normal physiologic function. The urgency of treatment is determined by the affected organ(s), the pace of symptom progression, and the physiologic stability of the patient. Uncontrolled infection may trigger an excessive immune response and dysregulated coagulation; localized infection may evolve progressively into sepsis (including organ failure according to the SEPSIS-3 definitions) or septic shock. Failure of therapy for surgical infections may reflect inadequate antibiotic therapy or failed SC; the latter should prompt consideration of immediate surgical re-intervention to mitigate worsening organ dysfunction, chronic critical illness, or death. If achieved by the index intervention, SC not only improves patients’ outcomes but also reduces antibiotic selection pressure by allowing a short course of antibiotic therapy [10, 11]. SC generally involves drainage of abscesses or infected fluid collections (whether by percutaneous or open drainage), debridement of necrotic or infected tissues, and definitive control of the source of contamination [12].

**Fig. 1:**
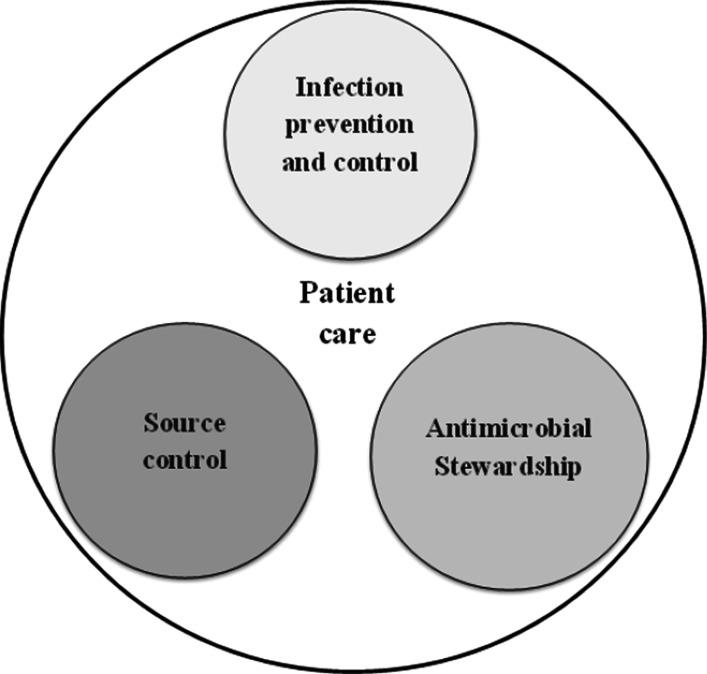
Patient care

AS, IPC, and SC are strictly interconnected and synergistic. Increased concern about the correct management of infections and increasing incidences of AMR in acute care facilities worldwide make multidisciplinary approaches necessary, reinforcing the concept that each aspect brings a particular contribution to patient care (Fig. 1). Successful prevention and management ensembles depend on HCWs behaviours and the organizational characteristics of acute health care facilities promoting behavioural change. This study was conducted to evaluate the organization of acute care facilities around the world in preventing and managing infections in surgery, to support proposed acute health care facilities' organizational standards.

## Methods

We conducted a cross-sectional electronic survey in order to: (1) investigate the organizational characteristics of acute care facilities worldwide in preventing and managing infections in surgery; (2) assess participants’ perception regarding ICP measures, antibiotic prescribing practices, and SC; (3) describe awareness about the global burden of AMR and IPC measures; and (4) determine the role of the coronavirus disease 2019 (COVID-19) pandemic on said awareness.

The population target was represented by the HCWs registered in the database of the Global Alliance for Infections in Surgery (GAIS). A total of 1432 HCWs were contacted via e-mail with an invitation letter and a survey link (Google Docs, Alphabet Inc., Mountain View, CA, USA). The survey was open for one month, between May 22, 2021, and June 22, 2021. Three reminders were sent, after 7, 14, and 21 days. The self-administered questionnaire (Additional File 1) was designed by a multidisciplinary team of investigators (including a surgeon, an epidemiologist, and an infectious disease physician) and was piloted among three physicians for face and content validity. The survey was written in English. Participation was voluntary but not anonymous; however, the confidentiality of respondents and their choices was ensured by de-identifying responses prior to data analysis. No incentives for participation were given. Data were automatically entered into an Excel database (Microsoft Corporation, Redmond, Washington, USA).

Published recommendations for the development and implementation of web-based surveys were applied to the design of our questionnaire [13, 14]. The self-administered questionnaire started with a characterization of the participants’ professional profiles, such as country, profession, years of experience, and membership of an IPC team or an AS team. Characteristics about working setting were also collected: type of hospital, hospital inpatient beds, existence and characteristics of the IPC team and the AS team, the existence of IPC measures; implementation and characteristics of local protocols for surgical antibiotic prophylaxis, and for therapy of surgical infections; existence and characteristics of surveillance systems for preventing health care associated infections (HAIs), and for monitoring SSIs; characteristics of laboratory testing; availability of periodic reports on local (AMR) data; availability and characteristics of radiological tools for emergency surgery; availability of a dedicated operating room for emergency surgery; and existence of surgical intensive care unit (ICU).

The questionnaire included 13 questions about participants’ perception regarding respect of IPC precautions, antibiotic prescribing practices, and principles of source control in their hospitals. Moreover, existence of teamwork, patient safety, and evidence-based medicine (EBM) culture were surveyed, as well as participants’ perception regarding HCWs role, their education and motivation. Finally, the last three questions investigated the participants’ awareness of the global burden of AMR, and the role of COVID-19 pandemic in raising awareness about IPC and appropriate antibiotic prescribing practices. Questions about participants’ perceptions were ranked with response options from 1 (very unrespected/unimportant/uninvolved/unaware) to 10 (very respected/important/involved/aware).

Based on the survey results, the investigators developed 15 statements for several questions regarding the prevention and management of infections in surgery. Agreement on the statements was reached by an Internet-based survey (using Google Docs). Statements were approved with an agreement of ≥ 80%. The statements may be the starting point for designing future evidence-based recommendations about infection prevention and management in surgery.

### Statistical analysis

Descriptive analysis for categorical variables is presented as frequency and percentage or median with interquartile range (IQR). Data regarding participants’ perceptions and awareness were collapsed into four categories as follows: 1–4, not respected/important/involved/aware; 5–6, slightly respected/important/involved/aware; 7–8, respected/important/involved/aware; 9–10, very respected/important/involved/aware. Categorical data were compared by chi-square or Fisher exact tests, as appropriate, using Stata 11 software (StataCorp, College Station, TX, USA). All tests were two-sided; p-values < 0.05 were considered significant.

## Results

### Baseline data: Coverage, response rate, professional profile, and working setting

Three hundred four respondents from 72 countries returned a questionnaire, with an overall response rate of 21.2%. Participants’ professional profiles and working settings are described in Table 1.

**Table 1:** Participants’ professional profile and working setting (304 participants)

Characteristics	*N* (%)
WHO Region classification	
European Region	198 (65.1)
Region of the Americas	29 (9.5)
South-East Asia Region	25 (8.2)
Easter Mediterranean Region	23 (7.6)
Africa Region	20 (6.6)
Western Pacific Region	9 (3.0)
Profession	
Surgeon	216 (71.1)
Infection diseases specialist	29 (9.5)
Infection control specialist	16 (5.3)
Intensivist or anaesthesiologist	14 (4.6)
Microbiologist	11 (3.6)
Epidemiologist or PH specialist	9 (3.0)
Other profession	9 (3.0)
Years of experience	
Less than 5 years	56 (18.4)
5–10 years	62 (20.4)
11–15 years	54 (17.8)
16–20 years	38 (12.5)
More than 20 years	94 (30.9)
Type of hospital	
University hospital	190 (62.5)
Community teaching hospital	62 (20.4)
Community hospital	43 (14.1)
Other	9 (3.0)
Hospital setting	
Urban	272 (89.5)
Suburban	25 (8.2)
Rural	7 (2.3)
Hospital inpatient beds	
≤ 100	10 (3.3)
101–500	109 (35.9)
501–1000	106 (34.9)
≥ 1000	79 (26.0)

*WHO* World Health Organization, *PH* public health

### IPC and AS teams: characteristics

Two hundred seventy-five (90.4%) participants stated their hospital had a multidisciplinary IPC team; 114 (114/275, 41.5%) declared they were currently team members. The median number of professionals comprising the IPC team was 6 [IQR 4–7]. Furthermore, 209 (68.8%) participants declared their hospital had a multidisciplinary AS team; 91 (91/209, 43.5%) stated they were currently team members. The median number of professionals comprising the AST was 5 [IQR 3–7].

In 204 hospitals (67.1%), both the IPC and AS teams were present, whereas, in 28 hospitals, no such teams were reported (9.2%). Two hundred forty-two (79.6%) participants declared to have at least one surgeon with interest or skills in surgical infections within their hospital. A surgeon with interest or skills in surgical infections was significantly less likely to be present in hospitals with < 100 inpatient beds (50.0%, *p* = 0.018) compared to hospitals with a larger number of inpatient beds (80.6%). Moreover, a surgeon with interest or skills in surgical infections was significantly less likely to be part of the IPC team in hospitals with < 100 inpatient beds (10.0%, *p* = 0.003) compared to larger hospitals (51.3%).

### Implementation of hygiene procedures, local protocols, and surveillance or monitoring systems

Local protocols for antimicrobial therapy of surgical infections were present in 233 hospitals (76.6%) (Table 2). Two hundred (200/233, 85.8%) survey participants declared the protocol for antimicrobial therapy included interventions to reduce duration of therapy, and 186 (186/233, 79.8%) stated the protocol advocated for alternative dosing tactics based on pharmacokinetic and pharmacodynamic principles. Furthermore, 211 (211/254, 83.1%) participants stated all hospital wards to have local protocols that included discontinuation of prophylaxis in the post-operative period. A surgical antibiotic prophylaxis protocol was significantly less likely to be implemented in hospitals < 100 inpatient beds compared with hospitals with a larger number of inpatient beds (60.0% vs. 91.8%, *p* < 0.001). No other statistically significant differences in implementation of local protocols and surveillance or monitoring systems were found as defined by World Health Organization (WHO, Geneva, Switzerland) region classification or work settings.

**Table 2:** Implementation of hygiene procedures, local protocols, and surveillance or monitoring systems (304 participants)

Implementation of hygiene procedures, local protocols, and surveillance or monitoring systems	All hospital wards [*n* (%)]	Some hospital wards [*n* (%)]	No hospital wards [*n* (%)]
HWDs of alcohol-based hand rub at the POC	273 (89.0)	28 (9.2)	3 (1.0)
Alcohol-based solutions for surgical site preparation	209 (68.7)	37 (12.2)	58 (19.1)
Protocol on prevention of specific HAIs	261 (85.9)	24 (7.9)	19 (6.2)
Protocol for surgical antibiotic prophylaxis	254 (83.6)	22 (7.2)	28 (9.2)
Protocol for AMT for surgical infections	233 (76.6)	0	71 (23.4)
Surveillance systems for SSIs	188 (61.8)	45 (14.8)	71 (23.4)
Monitoring systems of used antimicrobials	155 (51.0)	76 (25.0)	73 (24.0)
Systematic reports about AMR data	178 (58.5)	58 (19.1)	68 (22.4)

*HWDs* hand wash dispensers, *POC* point of care, *HAIs* health care associated infections, *AMT* antimicrobial therapy, *SSIs* surgical site infections, *AMR* antimicrobial resistance

### Other hospital characteristics

Two hundred nineteen participants (72.0%) declared their hospitals to have systems for rapid laboratory testing, 279 (91.8%) had 24-h computed tomography capability, 201 (66.1%) had 24-h interventional radiology capability, 250 (82.2%) had dedicated operating room facilities for emergency surgery, and 247 (81.2%) had a surgical ICU.

### Participants’ perception of respect of basic measures in their hospital

The majority of participants (244, 80.3%) stated that basic IPC measures are very respected or respected in their hospitals, whereas 99 (32.6%) declared basic antibiotic prescribing practices are slightly or not respected in their hospitals (Table 3). Basic antibiotic prescribing practices were more respected in hospitals with AS teams (75.6% vs. 49.5% *p* < 0.001), and in hospitals with implemented protocols for antimicrobial therapy of surgical infections (74.6% vs. 38.3%, *p* < 0.001).

**Table 3:** Participants’ perception of respect of basic measures in their hospital

Questions	Very respected [*n* (%)]	Respected [*n* (%)]	Slightly respected [*n* (%)]	Not respected [*n* (%)]
How much are basic infection prevention and control precautions respected in your hospital?	120 (39.5)	124 (40.8)	48 (15.8)	12 (3.9)
How much are basic antibiotic prescribing practices respected in your hospital?	81 (26.6)	124 (40.8)	71 (23.4)	28 (9.2)
How much are basic principles of source control respected in your hospital?	120 (39.5)	110 (36.2)	54 (17.8)	20 (6.5)

### Participants’ perceptions of importance of teamwork, patient safety, and EBM culture in their hospitals

**Fig. 2:**
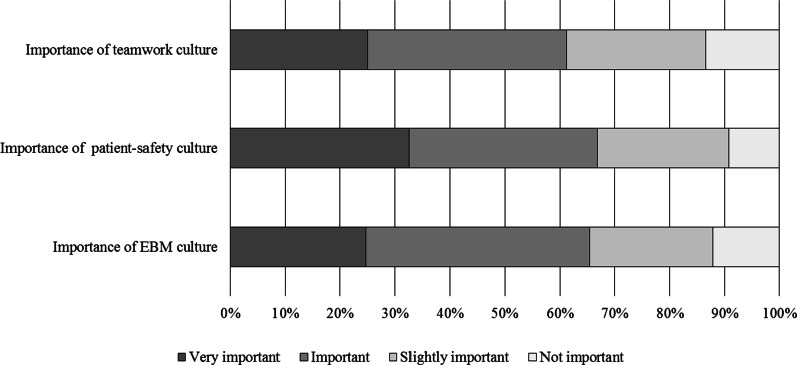
Participants’ perceptions of importance of teamwork, patient safety and evidence-based medicine culture in their hospitals. *EBM* Evidence-based medicine

The majority of participants stated the culture of teamwork (186, 61.2%), patient safety culture (203, 66.8%), and culture of EBM (199, 65.5%) are considered very important or important in their hospitals (Fig. 2). Greater importance was ascribed to teamwork culture (68.9% vs. 44.2%, *p* < 0.001), patient safety culture (71.8% vs. 55.8%, *p* = 0.006), and EBM culture (74.2% vs. 46.3%, *p* < 0.001) by respondents working in hospitals with an AS team.

### Participants’ perceptions of the importance of HCWs and patients’ and families’ roles in their hospital

HCWs participation in IPC was considered very important or important by 181 (59.5%) of respondents (Table 4). In hospitals with less than 100 inpatient beds, HCWs involvement in IPC was significantly more important (90.0% vs. 58.5%, *p* = 0.046) compared to hospitals with a larger number of inpatient beds. Furthermore, greater HCWs involvement in IPC was observed among respondents working in hospitals with IPC teams (62.2% vs. 34.5%, *p* = 0.004) and AS teams (67.0% vs. 43.2%, *p* < 0.001.

**Table 4:** Participants’ perceptions of the involvement of HCWs and patients’ and families’ roles in their hospital

Questions	Very involved [*n* (%)]	Involved [*n* (%)]	Slightly involved [*n* (%)]	Not involved [*n* (%)]
How much are health care workers involved in infection risk in your hospital?	87 (28.6)	94 (30.9)	75 (24.7)	48 (15.8)
How much are patients and families involved to help and support the journey to safer infections management in your hospital?	29 (9.6)	77 (25.3)	94 (30.9)	104 (34.2)

Patient and family involvement in IPC management was considered to be slightly or not important in their own hospital by the majority of respondents (198, 65.1%), and greater participation of loved ones was detected among respondents working in hospitals with IPC teams (37.1% vs. 13.8%, *p* = 0.012) and AS teams (40.7% vs. 22.1%, *p* = 0.002).

### Participants’ perceptions about education and motivation in their hospitals

Respectively, 172 (56.6%) and 180 (59.2%) participants stated the contribution of education and motivation of HCWs to the design and delivery of safe systems for infections management was very important or important in their hospital. A greater importance (very important or important) about education of HCWs was significantly more likely to be present in hospitals with an AS team compared to hospitals without (39.0% vs, 64.6%, *p* < 0.001). Similarly, greater importance (very important or important) ascribed to motivation of HCW was significantly more likely to be present in hospitals with an AS team compared to hospitals without (48.4% vs. 64.1%, *p* = 0.010).

### Participants’ awareness about global burden of AMR in their hospitals

Over half of the surveyed participants (166, 54.6%) stated the awareness of the global burden of AMR was very widespread or widespread among health care workers in their own hospitals. Specifically, greater awareness of the global burden of AMR (very important or important) was observed among participants working in hospitals with implemented SSI surveillance systems (63.1% vs. 26.8%, *p* < 0.001), monitored antimicrobial use (62.8% vs. 28.8%, *p* < 0.001), and reports about AMR data (63.1% vs. 25.0%, *p* < 0.001).

### Participants’ perception of the role of COVID-19 pandemic

**Fig. 3:**
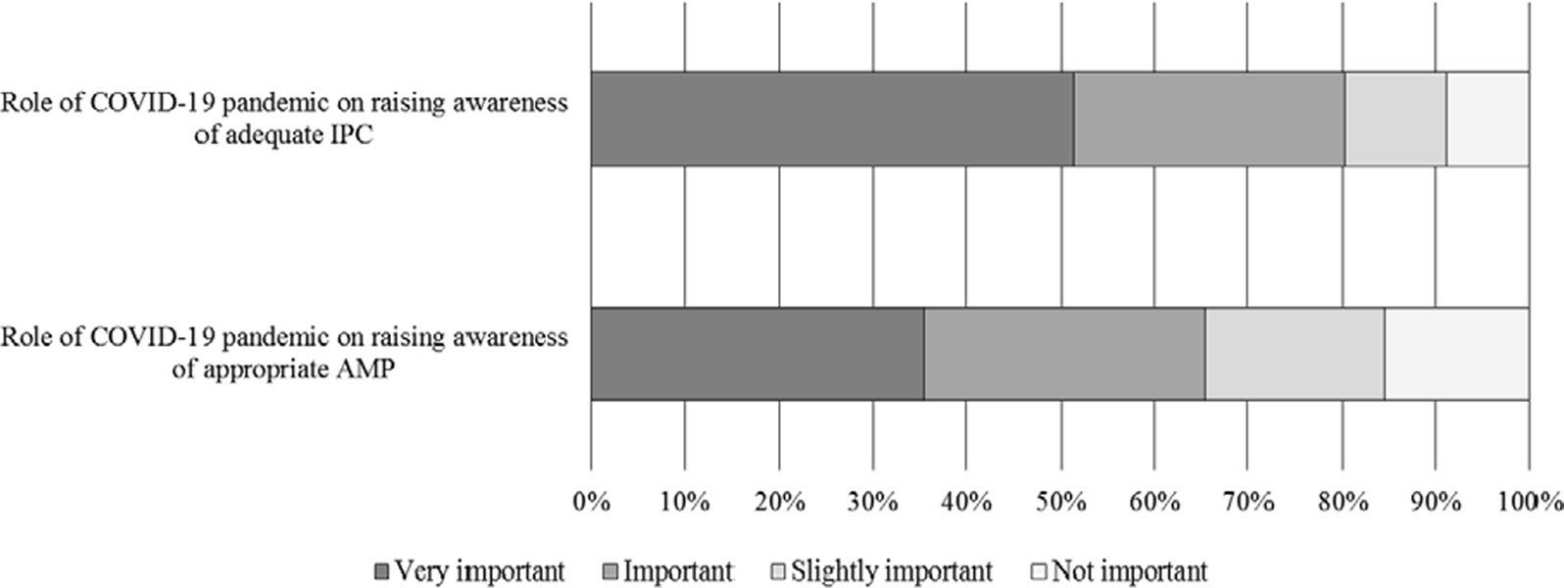
Participants’ perception of the role of COVID-19 pandemic. *IPC* Infection prevention and control, *AMP* antimicrobial prescription

The COVID-19 pandemic was considered by 244 (80.3%) respondents as a very important or important factor in raising HCWs awareness of the IPC programs in their own hospital, and by 199 (65.5%) in increasing consciousness regarding appropriate antibiotic prescribing (Fig. 3). No statistically significant differences in these perceptions were found according to the WHO region classification, the work setting, or the professional profile. Nonetheless, HCWs working in hospitals with multidisciplinary AS teams declared that the COVID-19 pandemic had a very important or important role in raising their awareness both on adequate IPC (85.2% vs. 69.5%, *p* = 0.001), and on appropriate antibiotic prescribing (71.3% vs. 52.6%, *p* = 0.001).

## Discussion

The primary goal of IPC programs is to prevent the acquisition and dissemination of HAIs within health care facilities. IPC programs should always include policies, procedures, and activities designed to prevent or reduce the spread of HAIs within health care facilities. Different hospital disciplines are typically involved in IPC programs, making collaboration and teamwork essential. IPC teams are effective in improving patients’ clinical outcomes, and cost-effective in providing important cost savings [15]. Raising awareness of IPC measures to stakeholders is a crucial factor in changing behaviour. The data of our survey demonstrated that the median number of professionals comprising the IPC team was 6 [IQR 4–7], including microbiologists (72.4%), infectious diseases specialists (70.2%), nurses (68.4%), hospital pharmacists or pharmacologists (67.6%), surgeons (56,7%), infection control specialists (56.4%), intensivists or anaesthesiologists (48.7%), hospital administrators (45.8%), epidemiologists (38.9%), public health specialists (30.2%), and emergency medicine specialists (18.2%).

Preventing SSIs is a priority for all surgical departments around the world. Bacteria are becoming increasingly resistant to antibiotics. SSIs are among the most common HAIs, making SSI prevention especially important. SSIs are associated with longer post-operative hospital stays, and higher attributable morbidity and mortality. SSIs prevention requires integrating a range of measures before, during, and after surgery. Both the WHO and the U.S. Centers for Disease Control and Prevention (CDC) have published guidelines for preventing SSIs [16–18]. The 2016 WHO Global guidelines for preventing SSIs are evidence-based, including systematic reviews presenting additional information supporting actions to improve practice [17, 18]. The guidelines include 13 recommendations for the pre-operative period and 16 for preventing infections during and after surgery, ranging from simple precautions such as ensuring that patients bathe or shower before surgery, effective skin disinfection for patients and surgical teams, guidance on when and for how long to use prophylactic antibiotics, and which sutures to use. According to the WHO Global guidelines, the use of alcohol-based solutions for the surgical site preparation is a strong recommendation and may be considered an important process indicator for SSIs prevention. In our survey, 68.7% of participants stated that alcohol-based solutions for surgical site preparation were used in all wards, while 12.2% in some wards, and 19.1% stated that alcohol-based solutions were not used.

The availability of guidelines is essential to provide a robust framework to support good clinical practice [17, 18]. Guidelines for the prevention of HAIs, including SSIs, have been published in recent years. Despite the clear evidence, compliance is uniformly poor, and significant difficulties arise when introducing evidence and clinical guidelines into routine daily practice.

Notably, guidelines alone are not sufficient to ensure adoption and the implementation of their principles and findings. Local adaptation is a prerequisite for successful guideline adoption and adherence. One way to engage HCWs in guideline development and implementation is to translate recommendations into a protocol or pathway that specifies and coordinates responsibilities for particular actions and timing among multidisciplinary team members in an acute care facility. Surveillance and outcomes assessment are crucial to monitor adherence with guideline recommendations. The results of our survey demonstrated that a protocol on HAIs prevention was in place in all hospital wards in the vast majority of acute facilities (85.9%), and only in 6.2% a protocol on HAIs prevention was not in place.

Hand hygiene is an important indicator of safety and quality of care in any health care setting. There is substantial evidence demonstrating the correlation between good hand hygiene practices and low HAI rates [19]. Failure to perform appropriate hand hygiene is considered the leading cause of HAIs and the spread of MDRO and has been recognized as an important contributor to outbreaks. There is convincing evidence that improved hand hygiene through multimodal implementation tactics can reduce HAI rates. In addition, several studies showed a sustained decrease in the incidence of MDRO isolates and patient colonization following the implementation of improved hand hygiene [19]. The point of care may be the starting point for an implementation program for hand hygiene, but an effective program of hand hygiene functions facility-wide with the participation of all HCWs. Most participants (89%) stated that hand washes dispensers of alcohol-based hand rub at the point of care were in place.

It is widely acknowledged that surveillance systems allow the evaluation of the local burden of HAIs and AMR and contribute to the early detection of HAIs and new patterns of AMR, including the identification of clusters and outbreaks. HAI surveillance is a challenging task as well because it requires particular expertise to analyse and assess epidemiologic data as to its quality and interpretation to tailor intervention and prevention measures. In our survey, participants stated that surveillance systems for SSIs were in place in all wards in 61.8% of acute care facilities, while in 23.4% were not in place. Systematic reports about AMR data were in place in all wards in 58.5% of acute care facilities, while in 22.4% were not in place.

AS programs have been promoted to optimize antimicrobial usage and patient outcomes and reduce the prevalence of AMR. However, the best tactics for an AS program are not definitively established and identifying optimal efforts to impact system change has been challenging. As programs are likely to vary based on local culture, available antibiograms, policy and routine clinical practice, and probably on resources [1]. Many hospitals remain without formal programs, and those that do continue to struggle to gain acceptance. Restriction of prescribing may be effective at controlling use but raises issues of prescriber autonomy and requires a large commitment of resources, including personnel. Multidisciplinary collaboration within health systems is mandatory to ensure that prophylactic, empiric, and directed use of antimicrobial agents results in optimal patient outcomes in the current era of AMR.

Every hospital worldwide should utilize existing resources to create an effective multidisciplinary team for AS [20]. Preferred aspects of AS programs include comprehensive collaboration among various specialties within a health care institution. The AS program is generally coordinated by infectious diseases specialists, whether physicians or pharmacists. Pharmacists with advanced training or longstanding clinical experience in infectious diseases should be key participants in the design and implementation of AS interventions. Infection control specialists and hospital epidemiologists should coordinate efforts to monitor and prevent HAIs and analyse and report “real-time” data to prevent infections, reduce antimicrobial use, and minimize the spread of AMR. Microbiologists should actively guide the proper use of tests and the flow of laboratory results, including periodic reports on AMR data within the facility, so as to allow the multidisciplinary team to determine the ongoing burden of AMR in the hospital. Moreover, timely and accurate reporting of antimicrobial susceptibility test results allows de-escalation to more appropriate targeted therapy and may help reduce broad-spectrum antimicrobial use.

Surgeons with expertise in surgical infections and surgical anatomy, when involved in AS programs, may audit antibiotic prescriptions, provide feedback to the prescribers, integrate best practices of antimicrobial use among surgeons, and act as champions among colleagues. Intensivists have a crucial role in treating MDROs of critically ill patients and thus are at the forefront of successful AS programs. Nurses are crucial for maintaining patient safety and monitoring the consequences of antimicrobial therapy. The engagement of hospital administration is a key factor for both developing and sustaining of AS programs. Without adequate support from hospital administration (AS programs do not generate revenue), programs will be inadequate or inconsistent.

In our survey 68.8% of participants stated that their hospital had a multidisciplinary AS team; 43.5% of respondents stated they were currently members of it. The median number of professionals working inside the AST was 5 [IQR 3–7], mainly represented by infectious diseases specialists, microbiologists, hospital pharmacists or pharmacologists, and infection control specialists.

Especially in resource-poor-settings, the IPC and AS teams can optimize bidirectional communication and collaborate in sharing resources and personnel. Data review, monitoring and reporting, and interventions such as audit and feedback and education are integral processes to both AS and IPC. Integrating these activities can make for more efficient workflow for both programs.

AS policies should be based on international and national antibiotic guidelines and tailored to local microbiology and AMR patterns. Based on the guidelines and local formulary options promoted by the AS team, facility-specific treatment recommendations can guide clinicians in antibiotic selection and duration of therapy. Standardizing and monitoring a shared protocol of surgical antibiotic prophylaxis is a logical first step in developing an AS program.

Local protocols for antimicrobial therapy of surgical infections were present in the majority of the hospitals (76.6%). Most participants (85.8%) declared the protocol for antimicrobial therapy included interventions to reduce the duration of therapy, while 79.8% stated the protocol advocated for alternative dosing tactics based on pharmacokinetic and pharmacodynamic principles. It is important to observe that 83.1% of participants stated all hospital wards have local protocols that included discontinuation of prophylaxis in the postoperative period. A surgical antibiotic prophylaxis protocol was significantly less likely to be implemented in hospitals with < 100 inpatient beds compared with hospitals with a larger number of inpatient beds (60.0% vs. 91.8%, *p* < 0.001).

Pharmacy’s contribution to AS programs has evolved substantially during the twenty-first century. Although infectious diseases specialist physicians and microbiologists have been responsible conventionally for providing advice on clinical management of infected patients, many pharmacists in clinical practice have now established roles complementing the expertise in multidisciplinary antimicrobial stewardship teams. Pharmacists’ responsibilities for AS include promoting the optimal use of antimicrobial agents. Typical interventions include patient-specific recommendations on optimization or de-escalation of antimicrobial therapy; and implementation of policies, education, therapeutic drug monitoring, and participation in AS ward rounds. Antibiotics are prescribed in up to one-third of hospital inpatients, often inappropriately, and more than two-third of critically ill patients are on antibiotics at any given time during hospitalization. Antibiotic use is one of the most direct and important parameters to assess the impact that an AS program has on a hospital and its patient population [21], although AMR and clinical outcomes are also important measures. Antimicrobial use (consumption) is a commonly used measures and is described by defined daily dose (DDD) or days of therapy (DOT), usually normalized per 1000 patient-days. The results of the survey demonstrated that monitoring systems of used antimicrobials were in place in all wards in just over half of acute care facilities.

Some of the most common clinical conditions that surgeons manage are infectious in nature. Additionally, HAIs such as SSIs, CA-UTIs, and HABP/VABP are among the most common complications surgeons face in clinical practice. Therefore, compliance with IPC measures and AS practices is integral to good clinical practice. However, both IPC and AS practices among surgeons are often inadequate. Surgeons are at the forefront in preventing infections in that they are responsible for many health care processes that impact the risk of HAIs and their prevention. Surgeons are also at the forefront of infection management of surgical patients, achieving prompt source control and providing adequate antibiotic therapy. In this context, surgeons’ participation in multidisciplinary efforts to improve surgical quality is crucial, including efforts to increase the evidence base [22, 23].

Increasing knowledge alone may be insufficient and ineffective unless education is continuous and interactive, including discussions of evidence, achievement of local consensus, and peer feedback on performance. Identifying a local opinion leader “champion” is important to facilitate integration of best clinical practices and encourage colleagues to change behaviours. Surgeon champions may provide feedback to prescribers and lead by implementing change personally, interacting directly with the AS team and the IPC team. The majority of participants stated to have at least one surgeon with an interest or skills in surgical infections within their hospital department. A surgeon with an interest or skills in surgical infections was significantly less likely to be present in hospitals with less than 100 inpatient beds (50.0%, *p* = 0.018) compared to hospitals with a larger number of inpatient beds (80.6%). Moreover, a surgeon with an interest or skills in surgical infections was significantly less likely to be part of the IPC team in hospitals with less than 100 inpatient beds (10.0%, *p* = 0.003) compared to larger hospitals (51.3%).

Appropriate SC is of utmost importance in managing complicated intra-abdominal infections and soft-tissue infections, Furthermore, adequate SC can also shorten the course of antibiotic therapy. The adequacy of SC is unrelated to appropriate antibiotic administration. Although each is an independent predictor of mortality, antibiotic therapy may have no effect without adequate SC. The level of urgency of treatment is determined by the affected organ(s), the relative speed at which clinical symptoms progress, and the underlying physiological stability of the patient. The challenges of multiple patients requiring emergency surgery or of limited resource availability highlight the importance of triage patients according to anatomic diagnosis and physiologic state. An expedient diagnosis of infection that requires emergent source control is crucial for patients with sepsis or septic shock; the source control intervention must be implemented as soon thereafter as is medically and logistically practical. Delays of as little 6 h have been associated with increased mortality [24–26]. Two hundred fifty (250/304 82.2%) participants stated that a dedicated operating room for emergency surgery was available 24 h a day.

Education spans all domains of health service delivery and is relevant to all health care workers, ranging from frontline workers to administrative management. Education of all health professionals in preventing and managing infections should begin at the undergraduate level and be supplemented with further training throughout the postgraduate years. Hospitals are responsible for educating clinical staff about IPC programs. According to available resources, education programs such as academic detailing, consensus building, and educational workshops should be implemented in each hospital worldwide.

Respectively 56.6% and 59.2% of participants stated the contribution of education and motivation of HCWs to the design and delivery of safe systems for infections management was very important or important in their hospital. A greater importance about the education of HCWs was significantly more likely to be present in hospitals with an AS team (64.6%, *p* < 0.001) compared to hospitals without (39.0%).

Effective teamwork in health care delivery can have an immediate and positive impact on patient safety. Health care teams that communicate effectively reduce the potential for human error, resulting in enhanced patient safety and improved clinical performance. Implementation research has demonstrated that best practice interventions are most effective when applied by teams that support the translation of evidence and guideline recommendations into practice, intending to change HCW behaviour. The majority of participants stated the culture of teamwork was considered very important or important in their hospitals. Patient safety is a crucial component of health care quality and is related to preventing and managing infections. Patient safety is a serious global public health issue that is defined as the prevention of harm to patients, with an emphasis on a culture of safety that involves health care professionals, organizations, and patients in a collaborative system of care delivery that prevents errors and learns from errors that do occur. In our survey, most participants stated that the culture of patient safety was very important or important in their hospitals.

Finally, many authors highlighted the need to increase patient involvement in IPC implementation in health care settings. Patient involvement in IPC may ensure a more patient-centred health care prioritising their needs and empowering them to take control of their own IPC [27, 28]. Patients and family involvement in IPC management was considered to be slightly or not important in their own hospital by the majority of respondents (65.1%). Although the low importance among respondents of patient involvement in IPC, both patients and HCWs should jointly advocate for a culture of patient involvement in reducing the burden of HAIs [27]. It would require changes to the organisational cultural model in which HCWs tend to control their organisation and also play an “authoritarian” role over patients [28]. The required cultural changes should imply a reversion in the relation between HCWs and patients and the need to put patients in a responsible and protagonist role as experts in their own care and IPC, rather than being passive participants and observers of HCWs’ behaviours.

This study has several limitations: a response rate of just 21.2% should be considered a response bias, and it is possible that non-participating HCWs may have been less interested in surgical infections than the participants and therefore it is possible that results are biased towards a better picture than it actually is. According to specialty, no stratification or sampling was pre-planned to ensure that all stakeholders were adequately represented and the questionnaire was self-reported. The major strength of the study is its multinational (global) and multidisciplinary approach, to our best knowledge the first in this setting. Thus, our survey provides a benchmark to all interested stakeholders; it can be repeated over time to explore if better uniformity on a global platform of health care environments would develop in the future, and may be used to build consensus around the best practices in the field of prevention and management of surgical infections a future project.

## Conclusions

Adequacy of prevention and management of infections depends on both HCWs behaviours and organizational characteristics of acute health care facilities to support best practices and promote behavioural change. Patient involvement in the implementation of IPC is still little considered. A debate on how operationalising a fundamental change to IPC, from being solely the HCWs responsibility to one that involves a collaborative relationship between HCWs and patients, should be opened.

Based on the survey results, the authors shared 15 strongly suggested statements regarding the principal questions surrounding the prevention and management of infections in surgery that may be the starting point for future evidence-based recommendations.

1. An IPC program should be in place in each acute care facility around the world. It should be led by professionals trained in and dedicated to IPC; however, it should also include allied health care workers who are directly involved in infection prevention measures in their areas of clinical expertise (208 responses: agreement 98.1%).
2. Each acute health care facility around the world should implement, according to the available resources, measures aimed at reducing the risk of surgical site infections (SSIs) before, during, and after surgery (208 responses: Agreement 100%).
3. A local adaptation of evidence-based guidelines for the prevention of HAIs, according to available resources, should be developed and implemented in each acute health care facility around the world. The education and training of health care workers on the recommendations and the monitoring of adherence with guideline recommendations should be undertaken to achieve successful implementation (208 responses: Agreement 99%).
4. Hand hygiene is the cornerstone of IPC. When optimally performed, hand hygiene reduces HAIs and the spread of AMR. Poor compliance with hand hygiene practices remains a challenge for IPC practitioners all over the world. Hand hygiene should be guaranteed at the point of care in each acute health care facility worldwide, according to the available resources (208 responses: Agreement 100%).
5. Facility-based HAI surveillance, including AMR surveillance, should be performed to guide interventions and detect outbreaks, according to available resources. The collection and analysis of monitoring data should serve to identify vulnerabilities in the system, and serve as the basis for organizational improvement and risk reduction. Timely feedback of results to HCWs should be provided (208 responses: agreement 97.6%).
6. According to the available resources, an antimicrobial stewardship program should be in place in each acute health care facility worldwide. It should be led by professionals trained in antimicrobial stewardship; however, it should also include allied health care workers directly involved in prescribing antibiotics in daily clinical practice (208 responses: Agreement 98.6%).
7. Local adaptation of evidence-based guidelines for appropriate antibiotic prescribing practices, according to local epidemiology and according to the available resources, should be developed and implemented in each acute health care facility around the world. The education and training of relevant health care workers on these guidelines and monitoring of adherence with their recommendations should be undertaken to achieve successful implementation (208 responses: Agreement 99.5%).
8. Monitoring of antibiotic utilization along with antimicrobial resistance surveillance data and clinical outcome measures should be performed regularly (e.g. every three to six months), with results provided to all antibiotic stewardship program team members for review (208 responses: Agreement 97.1%).
9. Every surgeon should have a basic understanding of need for and approaches to preventing and managing infections. However, surgeons with special interest and knowledge in surgical infections should be incorporated into the infection control and antimicrobial stewardship teams and recognized as “champions” (208 responses: Agreement 95.2%).
10. Capability of performing source control procedures to treat surgical infections, including dedicated operating rooms, should be available 24 h a day in each acute health care facility around the world, in order to avoid unnecessary delays in treating time-dependent infections (208 responses: Agreement 95.7%).
11. Education in preventing and managing infections in surgery, by utilizing team- and task-based tactics, should be available for all health care workers in every acute health care facility around the world (208 responses: Agreement 99.5%).
12. Implementation of team work activities to improve clinical practices in preventing and managing infections in surgery are suggested for every acute health care facility around the world (208 responses: Agreement 98.6%).
13. A culture emphasizing patient safety should be a strategic goal of every acute health care facility around the world (208 responses: Agreement 99.5%).
14. According to the available resources, protocols for triage of patients requiring emergency surgery for surgical infections should be implemented in each acute health care facility around the world (208 responses: Agreement 97.6%).
15. Each acute health care facility around the world should organize local initiatives to improve sepsis-related mortality. These include hospital-based programs for sepsis prevention, early detection, and delivery of early treatment. As part of a hospital-wide program on sepsis, a dedicated “sepsis team” to evaluate patients with sepsis and septic shock may favourably influence outcomes (208 responses: Agreement 91.8%).

## Supplementary Information

**Additional file 1.** Questionnaire.

## Data Availability

Not applicable.

## Abbreviations

AMR: Antimicrobial resistance
AS: Antimicrobial stewardship
HAIs: Health care-associated infections
HCWs: Health care workers
IPC: Infection prevention and control
MDROs: Multidrug resistant organism
SC: Source control
SSIs: Surgical site infections
