# Supplementary material for: It is time to define an organizational model for the prevention and management of infections along the surgical pathway: a worldwide cross-sectional survey
Source: World J Emerg Surg. 2022 Mar 17;17:17. doi: 10.1186/s13017-022-00420-4 (PMC8928018; doi:10.1186/s13017-022-00420-4)
Supplement: Supplementary file 1 — Additional file 1. Questionnaire. [file 13017_2022_420_MOESM1_ESM.doc]

**Additional File 1. Questionnaire.**

**1. In which country are you currently working?**

Please, choose from the list.

**2. Which is your profession?**

Epidemiologist _1_

Infection control specialist _2_

Infection diseases specialist _3_

Intensivist or anestesiologist _4_

Microbiologist _5_

Public Health specialist _6_

Surgeon _7_

Other profession _____________________ _8_

**3. How many years of experience do you have?**

Less than 5 years _1_

5 – 10 years _2_

11-15 years _3_

16 – 20 years _4_

More than 20 years _5_

**4. What type of hospital are you working in?**

University Hospital _1_

Community Hospital _2_

Community teaching Hospital _3_

Other __________________ _4_

**5. What is the setting of your hospital?**

Urban _1_

Suburban _2_

Rural _3_

**6. How many inpatient beds has the hospital where you practice the most of your time?**

Less than 100 _1_

101-500 _2_

501-1000 _3_

More than 1000 _4_

**7. Does your hospital have a multidisciplinary infection prevention and control (IPC) team?**

Yes _1_

No _3_

**8. Are you currently a member of the IPC team?**

Yes _1_

No _3_

**9. Which professionals does the IPC team include?**

Emergency medicine specialist _1_

Epidemiologist _2_

Hospital Administrator _3_

Hospital pharmacist _4_

Hospital pharmacologist _5_

Infection control specialist _6_

Infection diseases specialist _7_

Intensivist or anestesiologist _8_

Microbiologist _9_

Nurse _10_

Public Health specialist _11_

Surgeon _12_

**10. Is there at least a surgeon with interest/skills in surgical infections in your surgical department of your hospital?**

Yes _1_

No _3_

**11. Does your hospital have hand wash dispensers of alcohol-based hand rub at the points of care?**

Yes _1_

Yes, but only in some wards _2_

No _3_

**12. Does your hospital have local protocols focusing on prevention of specific healthcare-associated infections?**

Yes _1_

Yes, but only in some wards _2_

No _3_

**13. Does your hospital have a protocol for surgical antibiotic prophylaxis?**

Yes _1_

Yes, but only in some wards _2_

No _37_

**14. Does the protocol for surgical antibiotic prophylaxis consider interventions to discontinue prophylaxis in the post-operative period?**

Yes _1_

Yes, but only in some wards _2_

No _3_

**15. Does your hospital use alcohol-based solutions for surgical site preparation?**

Yes _1_

Yes, but only in some wards _2_

No _3_

**16. Does your hospital have a surveillance system for surgical site infections?**

Yes _1_

Yes, but only in some wards _2_

No _3_

**17. Does your hospital have a system for root cause analysis/review of surgical site infections?**

Yes _1_

No _3_

**18. Does your hospital have a multidisciplinary antimicrobial stewardship team (AST)?**

Yes _1_

No _3_

**19. Are you currently a member of the AST team?**

Yes _1_

No _3_

**20. Which professionals does the AST team include?**

Emergency medicine specialist _1_

Epidemiologist _2_

Hospital Administrator _3_

Hospital pharmacist _4_

Hospital pharmacologist _5_

Infection control specialist _6_

Infection diseases specialist _7_

Intensivist or anestesiologist _8_

Microbiologist _9_

Nurse _10_

Public Health specialist _11_

Surgeon _12_

**21. Does your hospital have a protocol for antimicrobial therapy?**

Yes _1_

No _3_

**22. Does the protocol for antimicrobial treatment of surgical infections consider interventions to reduce the duration of therapy?**

Yes _1_

No _3_

**23. Does the protocol for antimicrobial treatment of surgical infections advocate for alternative dosing strategies based on pharmacokinetic/pharmacodynamic principles?**

Yes _1_

No _3_

**24. Does your hospital have systems for rapid laboratory testing?**

Yes _1_

No _3_

**25. Does your hospital perform a monitoring system of used antimicrobials?**

Yes _1_

Yes, but only in some wards _2_

No _3_

**26. Does your hospital periodically carry out systematic reports about resistance data?**

Yes _1_

Yes, but only in some wards _2_

No _3_

**27. Did your hospital carry out structural interventions finalized to improve antimicrobial stewardship programs and infection prevention and control programs in the last 5 years?**

Yes _1_

No _3_

**28. Does your hospital have a 24-hour CT scan for emergency cases?**

Yes _1_

No _3_

**29. Does your hospital have a 24-hour interventional radiology service for emergency cases?**

Yes _1_

No _3_

**30. Does your hospital have a dedicated operating room for emergency surgery?**

Yes _1_

No _3_

**31. Does your hospital have a surgical intensive care unit?**

Yes _1_

No _3_

**32. How much are basic infection control precautions respected in your hospital?**

Please, rank from 10 (very respected) to 1 (very unrespected).

**33. How much are basic antibiotic prescribing practices respected in your hospital?**

Please, rank from 10 (very respected) to 1 (very unrespected).

**34. How much are basic principles of source control respected in your hospital?**

Please, rank from 10 (very respected) to 1 (very unrespected).

**35. How much is teamwork culture important in your hospital?**

Please, rank from 10 (very important) to 1 (very unimportant).

**36. How much is patient safety culture important in your hospital?**

Please, rank from 10 (very important) to 1 (very unimportant).

**37. How much is evidence-based culture important in your hospital?**

Please, rank from 10 (very important) to 1 (very unimportant).

**38. How much are healthcare workers involved in infection risk in your hospital?**

Please, rank from 10 (very involved) to 1 (very uninvolved).

**39. How much are patients and families involved to help and support the journey to safer infections management in your hospital?**

Please, rank from 10 (very involved) to 1 (very uninvolved).

**40. How much does education of healthcare workers contribute to the design and delivery of safe systems for infections management in your hospital?**

Please, rank from 10 (very important contribution) to 1 (very unimportant contribution).

**41. How much does motivation of healthcare workers contribute to the design and delivery of safe systems for infections management in your hospital?**

Please, rank from 10 (very important contribution) to 1 (very unimportant contribution).

**42. How much is awareness of the global burden of antibiotic resistance widespread among healthcare workers in your hospital?**

Please, rank from 10 (very aware) to 1 (very unaware).

**43. How much has the COVID-19 pandemic raised your awareness of adequate infection prevention and control?**

Please, rank from 10 (very important role) to 1 (very unimportant role).

**44. How much has the COVID-19 pandemic raised your awareness of appropriate antibiotic prescribing practices?**

Please, rank from 10 (very important role) to 1 (very unimportant role).
